# Analgesic efficacy and serum ropivacaine concentration of postoperative programmed intermittent bolus infusion with serratus anterior plane block in patients undergoing minimally invasive cardiac surgery: A randomized, double-blind, controlled trial

**DOI:** 10.1007/s00540-025-03536-4

**Published:** 2025-07-01

**Authors:** Yuna Sato, Michio Kumagai, Yu Kaiho, Shigekazu Sugino, Tomohiro Sekine, Masataka Taguri, Hiroshi Inoue, Jun Ito, Yu Sato, Toshihiro Sato, Masamitsu Maekawa, Masanori Yamauchi

**Affiliations:** 1 Department of Anesthesiology and Perioperative Medicine, Tohoku University Graduate School of Medicine, 2-1, Seiryo-Machi, Aoba-Ku, Sendai, 980-8575 Japan; 2 Department of Anesthesiology, Japan Red Cross Ishinomaki Hospital, Ishinomaki, Japan; 3 Department of Health Data Science, Tokyo Medical University, Shinjuku City, Japan; 4 Department of Anesthesiology, Southern Tohoku General Hospital, Iwanuma, Japan; 5 Department of Anesthesiology, Sendai Kousei Hospital, Sendai, Japan; 6 Department of Pharmaceutical Sciences, Tohoku University Hospital, Sendai, Japan

**Keywords:** Serratus anterior plane block, Minimally invasive cardiac surgery, Postoperative analgesia, Programmed intermittent bolus infusion

## Abstract

**Purpose:**

Minimally invasive cardiac surgery (MICS) involves fewer complications than median sternotomy. However, difficulties in post-MICS analgesia can undermine these advantages. The serratus anterior plane block (SAPB), an effective analgesic for thoracic surgery, could benefit post-MICS analgesia using programmed intermittent bolus infusion (PIBI). We investigated whether PIBI with SAPB can reduce post-MICS fentanyl administration and evaluated its safety profile.

**Methods:**

This randomized, double-blind, controlled trial included 20 patients (age 20–80 years; Eastern Cooperative Oncology Group Performance Status 0–II; scheduled for elective MICS) randomly allocated to two groups (SAPB or control). All patients underwent preoperative SAPB with catheterization, followed by either 20 mL 0.25% ropivacaine or saline bolus every 6 h postoperatively. All patients received intravenous fentanyl via patient-controlled analgesia. Blood samples were collected 10, 20, 30, and 60 min after preoperative ropivacaine infusion; during and after cardiopulmonary bypass; and on postoperative days 1–5. The primary outcome was cumulative fentanyl consumption up to postoperative day 5. Secondary outcomes included numerical rating scale scores, rehabilitation preoperatively and postoperatively, postoperative nausea and vomiting, ropivacaine toxicity, and PIBI with SAPB complications.

**Results:**

After excluding three patients, data from 17 patients were analyzed. No significant difference in cumulative fentanyl consumption on postoperative day 5 was observed (SAPB: median [interquartile range], 512 µg [457–753] vs. control: 654 µg [439–982], P = 0.96). Serum ropivacaine concentration remained below the toxicity threshold.

**Conclusion:**

PIBI with SAPB did not reduce post-MICS fentanyl consumption, and serum ropivacaine concentration did not reach the toxicity threshold.

**Supplementary Information:**

The online version contains supplementary material available at 10.1007/s00540-025-03536-4.

## Introduction

Minimally invasive cardiac surgery (MICS) is used for procedures such as atrial septal defect repair, coronary artery bypass, and valve procedures. The 5–7 cm thoracotomy incision used in MICS, compared with traditional median sternotomy, may cause fewer complications and earlier patient recovery [1]. However, post-MICS pain in the lateral intercostal region may undermine these benefits due to possible rib extraction, muscle dividing, and intercostal nerve injury [2].

Postoperative pain management after cardiac surgery relies on opioids, which may induce respiratory depression, nausea, and vomiting, affecting early recovery [2]. Paravertebral block and thoracic epidural analgesia are gold-standard approaches for postoperative pain after thoracic surgery. However, neuraxial nerve block may cause epidural hematoma and neurologic deterioration in patients receiving anticoagulants or antiplatelet medication, or from heparinization during cardiopulmonary bypass (CPB) [3, 4].

Serratus anterior plane block (SAPB), the infusion of substantial local anesthetic either above or below the serratus anterior muscle, causes a blockade of the lateral cutaneous branches of the intercostal nerves [2]. SAPB can be performed in the same position as surgery and minimizes the risk of incompressible bleeding. Therefore, an SAPB catheter can be safely placed, even in patients undergoing cardiac surgery with heparinization. Several studies investigated the efficacy of single-shot and continuous SAPB for post-MICS pain management; however, the results are inconsistent [5–7].

A programmed intermittent bolus infusion (PIBI) is commonly used for continuous nerve block. PIBI with a paravertebral block, a compartment-based approach relying on the rostrocaudal spread of local anesthetic, as with SAPB, provides superior postoperative analgesia to continuous infusion in lung surgery [8]. Thus, PIBI with SAPB may be a useful approach for post-MICS pain management. Nevertheless, clinical trials have not investigated this. Furthermore, though SAPB requires a large dose and volume of local anesthetic, the serum concentration of the local anesthetic after SAPB remains unknown.

Therefore, we aimed to conduct a randomized, double-blind, controlled trial to assess the analgesic effect of PIBI with SAPB on post-MICS fentanyl consumption and evaluate the adverse effects and serum concentration of ropivacaine to ensure the safety of this approach. We hypothesized that PIBI with SAPB may improve post-MICS pain management.

## Methods

### Study design and patient enrollment

This randomized, double-blind, controlled trial was conducted in accordance with the Consolidated Standards of Reporting Trials (CONSORT) guidelines. After obtaining approval from the institutional review board of Sendai Kousei Hospital (approval number: 2–36) on September 16th 2020, the study was registered prior to patient enrollment at the University Hospital Medical Information Network (identification number: UMIN000041814; https://www.umin.ac.jp/ctr/index.htm; principal investigator: Yuna Sato; registration date: September 16th 2020). This study was carried out in accordance with the Declaration of Helsinki, and written informed consent was obtained from all patients.

This study was conducted at Sendai Kousei Hospital between September 2020 and February 2022. The inclusion criteria were as follows: elective patients, aged 20–80 years, who underwent MICS with a lateral thoracic approach and Eastern Cooperative Oncology Group Performance Status (ECOG PS) 0–II. The exclusion criteria were as follows: patients with history of thoracotomy or cardiac surgery, long-term opioid use, analgesic use for > 2 months, allergies to local anesthetics, liver failure, renal insufficiency, infection at the block site, pregnancy, and/or psychiatric diseases.

The patients were randomized into two equal groups (SAPB and control groups). Randomization was performed using a computer-generated randomization sequence (http://www.randomizer.org) by an investigator who was not involved in patient care or perioperative assessment. Allocation numbers were enclosed within opaque envelopes, accessible solely to pharmacists responsible for dispensing medications for postoperative PIBI. After admission to the hospital, blinded physical therapists evaluated the rehabilitation. The principal investigator was blinded and responsible for anesthesia and postoperative evaluation. Blinded physical therapists and ward nurses assisted with the evaluation.

### Intervention

None of the patients received any premedication. General anesthesia was induced using midazolam and remifentanil; muscle relaxation was achieved using rocuronium. Anesthesia was maintained using a total intravenous (IV) infusion of propofol. A right anterior thoracotomy was performed for aortic valve surgery, whereas a right mini thoracotomy was performed for mitral valve surgery and atrial septal defect repair. A port for endoscopy was inserted through the same or an adjacent intercostal space as that used for the surgical procedure.

SAPB was performed by the principal investigator, who was experienced in ultrasound-guided nerve blocks, after inducing general anesthesia and positioning the patient for surgery. Ropivacaine (3 mg/kg, maximum 150 mg) was used for the SAPB and catheter insertion. A 6–13 MHz linear probe (SonoSite SII; FUJIFILM Medical, Tokyo, Japan) was placed on the caudal rib of the surgical site near the middle axillary line in the sagittal plane. A 17-G Tuohy needle (Hakko Co., Nagano, Japan) was inserted. After contacting the rib, half the dose of ropivacaine was used to hydro-dissect the plane between the serratus anterior muscle and rib. The catheter was inserted into the hydro-dissected plane, and the other half-dose of ropivacaine was injected through the catheter. Following intensive care unit (ICU) admission, the PIBI pump (CADD®-Legacy PLUS; Smiths Medical, St. Paul, MN) was connected to the SAPB catheter. The SAPB group received a bolus of 20 mL of 0.25% ropivacaine every 6 h from ICU admission; the control group received a 20-mL saline bolus. This PIBI protocol was determined based on previous studies [8–10].

IV fentanyl (10 µg/kg) was administered after weaning from CPB, followed by IV patient-controlled analgesia (PCA) upon ICU admission. The PCA device (CADD®-Solis PIB; Smiths Medical) delivered 0.4 µg/kg/h background infusion and 20 µg on-demand bolus, with a 7-min lockout time.

All patients were extubated if they were awake or arousable, neurologically intact, hemodynamically stable with minimal or no inotropic support, no active bleeding, and had satisfactory arterial blood gas levels. Following extubation, the background infusion of fentanyl was stopped, and patients were managed using a standardized multimodal analgesic protocol based on routine clinical practice. This included loxoprofen 60 mg three times daily for patients without impaired renal function and acetaminophen 500 mg four times daily for those with renal impairment. The patients evaluated their pain at rest and on movement using an 11-point numerical rating scale (NRS; 0 = no pain, 10 = worst imaginable pain). Rescue analgesics (IV flurbiprofen 50 mg or IV acetaminophen 15 mg/kg [or 1,000 mg for patients weighing over 50 kg], depending on renal impairments) were administered when the pain score at rest was ≥ 4 and on patient request. If pain remained at NRS ≥ 4 despite rescue analgesia and the patient’s request, a background infusion of fentanyl (0.4 µg/kg/h) was reinitiated. The patients were treated with 10 mg metoclopramide for severe nausea or vomiting. PIBI with SAPB was continued until postoperative day (POD) 5. The incidence of ropivacaine toxicity was assessed based on symptoms, such as seizures, agitation, or loss of consciousness. Complications due to PIBI with SAPB, such as bleeding or hematoma, insertion site infection, and neuritis were recorded. We defined postoperative nausea and vomiting (PONV) as the use of antiemetic medication at least once during the study period.

Blood samples (6 mL) were collected at 10, 20, 30, and 60 min after preoperative ropivacaine infusion; during and after CPB; and at any time on PODs 1–5. The blood samples were collected from the indwelling arterial catheter until the catheter was removed, after which venipuncture was performed. The samples were centrifuged at 2,330 × *g* for 5 min at 4ºC and the serum samples were stored at -80ºC until assayed as a batch. Serum samples (5 µL) were deproteinized by adding 95 µL of 50% methanol and 350 µL of acetonitrile containing internal standard (IS). The mixture was vortexed and centrifuged at 14,000 × *g* for 5 min at 4ºC, and the supernatant was used directly for measurement.

Chromatographic separation was carried out using the Shimadzu Nexera HPLC System (Shimadzu, Kyoto, Japan) with an InertSustain Swift C18 column (50 × 2.1 mm i.d., 3 µm HP, GL Sciences, Tokyo, Japan). To determine the total ropivacaine concentrations, the column was eluted with a binary flow of mobile phases A and B at a flow rate of 0.4 mL/min. The mobile phases A and B consisted of a 10 mmol/L ammonium formate buffer in water (pH 3.6) and 0.1% formic acid in acetonitrile, respectively. The gradient program was as follows: 0–5.5 min, 5–55% B; 5.5–5.51 min, 55–100% B; 5.51–7.0 min, 100% B; 7.0–7.01 min, 100–5% B; 7.01–8.0 min, 5% B. Subsequently, 2 µL of the sample was injected, and the column temperature was maintained at 40ºC. Positive-ion electrospray tandem mass spectrometric analysis was performed using an API 5000 tandem mass spectrometer (SCIEX, Framingham, MA) at a unit resolution with selected reaction monitoring (SRM). For positive electrospray ionization, the monitored SRM transitions were *m/z* 275 > 126 and *m/z* 282 > 133 for ropivacaine and ropivacaine-d7 (IS), respectively. The curtain gas, collision gas, ion spray voltage, turbo gas temperature, nebulizer gas, and turbo gas were set to 15 psi, 6 psi, 4500 V, 700ºC, 70 psi, and 40 psi, respectively. The other tandem mass spectrometry parameters used in the SRM analysis are summarized in Supplementary Table 1. The data were acquired and analyzed using Analyst® software 1.5 (SCIEX). The assay was validated by spiking negative plasma with known concentration of ropivacaine (1.5–5000 ng/mL), with a limit of detection of 1 ng/mL and limit of quantification of 5 ng/mL. In accordance with Knudsen et al. [11], 4.3 µg/mL (arterial samples) and 2.2 µg/mL (venous) were used as the toxic thresholds of ropivacaine.

### Outcomes

The primary endpoint was cumulative fentanyl consumption up to POD 5. The secondary outcomes were: NRS at rest and on movement at the time of extubation and 1, 2, 6, 12, 24, 48, 72, and 96 h after extubation, serum concentration of ropivacaine, evaluation of rehabilitation at preoperative and PODs 1–5 (grip strength, chest expansion measurement, range of motion angle at the upper limb joint), Short Physical Performance Battery (SPPB) score and 6-min walk distance (preoperative and at the end of the study period), incidence of PONV, incidence of ropivacaine toxicity, and complications due to PIBI with SAPB.

### Statistical analysis

All statistical data were analyzed using EZR version 1.61 [12] (Jichi Medical University, Saitama, Japan). Continuous data were tested for normality using a histogram and the Shapiro–Wilk test. Normally distributed, non-normally distributed, and categorical data are presented as means (standard deviation), medians (interquartile range), and numbers (percentage), respectively. Since the data of the primary outcome was not normally distributed, we used the Mann–Whitney *U* test to compare this outcome between the SAPB and control groups. In a secondary analysis, non-normally distributed data (NRS scores, rehabilitation evaluation) were compared using the Mann–Whitney *U* test; Bonferroni correction was used for multiple testing. Normally distributed data (6-min walk distance) were compared using Student’s *t*-test. Categorical outcome data (PONV, SPPB score, ropivacaine toxicity, and complications) were compared using Fisher’s exact test. Two-tailed *P* < 0.05 (< 0.0083 when multiple comparisons were performed for the evaluation of rehabilitation) was considered statistically significant.

The sample size for this study was determined based on our institutional data. A retrospective analysis was conducted on six patients, including three who underwent SAPB with PIBI and three in the control group. Total fentanyl consumption from the end of surgery to ICU discharge, including continuous infusion until discontinued by nursing judgment and bolus doses administered in response to patient-reported pain, was calculated. The mean total fentanyl consumption was 730 µg in the SAPB group (n = 3) and 2005 µg in the control group (n = 3). For a 95% chance of detecting a significant difference (at the two-sided 5% level) with an assumed standard deviation of 600 and a 30% loss to follow-up, 10 patients (20 in total) in each group were required.

## Results

**Fig. 1:**
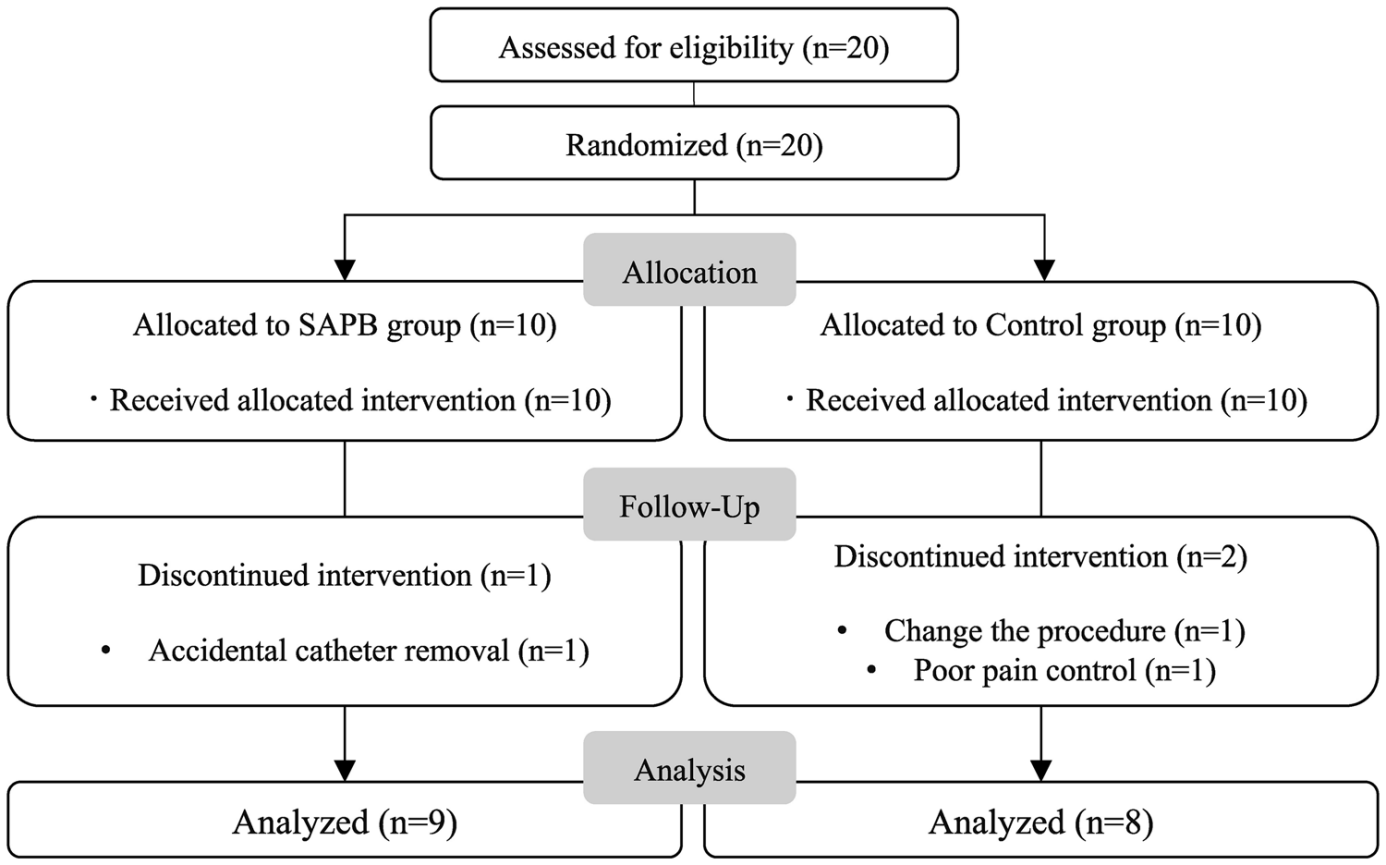
CONSORT flow chart for this study. *CONSORT* Consolidated Standards of Reporting Trials, *SAPB* serratus anterior plane block

Twenty patients were enrolled. Three patients were excluded from the analysis: one patient in the SAPB group who had an accidental catheter removal, one patient in the control group who was converted to median sternotomy, and one patient in the control group who declined to complete the study owing to poor pain control on POD 2. Seventeen patients were included in the final analysis: nine and eight patients in the SAPB and control groups, respectively (Fig. 1).

The baseline and intraoperative data showed no clinically significant differences between the two groups (Table 1). Most cases involved mitral valve plasty (78 vs. 50% in the SAPB and control groups, respectively).

**Table 1:** Baseline characteristics

		**Group**	
		**SAPB**	**Control**
N, cases		9	8
Age, mean (SD), years		57.7 (10.4)	55.3 (14.5)
Sex (female), n (%)		2 (22)	5 (63)
Height, mean (SD), cm		162.1 (8.0)	159.7 (6.9)
Weight, mean (SD), kg		59.7 (11.1)	61.7 (15.8)
ECOG PS, n (%)	0	2 (22)	1 (13)
	1	7 (78)	6 (75)
	2	0 (0)	1 (13)
Diagnosis, n (%)	AR	1 (11)	2 (25)
	AS	1 (11)	0 (0)
	ASD	0 (0)	1 (13)
	ASD, TR	0 (0)	1 (13)
	MR	7 (78)	4 (50)
Surgical procedure, n (%)	ASD	0 (0)	1 (13)
	ASD, TAP	0 (0)	1 (13)
	AVR	2 (22)	2 (25)
	MVP	7 (78)	4 (50)
SPPB, n (%)	11	0 (0)	2 (25)
	12	9 (100)	6 (75)
6-min walk distance, mean (SD), m		434.9 (41.8)	426.1 (46.7)
Measurement of chest expansion, mean (SD), cm		4.8 (2.0)	3.2 (1.6)
Grip strength in right hand, mean (SD), kg		37.7 (9.4)	28.1 (8.8)
Shoulder flexion ROM, median (IQR), °		175.0 [170.0, 180.0]	180.0 [168.8, 180.0]
Shoulder abduction ROM, median (IQR), °		170.0 [160.0, 170.0]	175.0 [170.0, 180.0]
Anesthesia time, mean (SD), min		356.6 (39.6)	342.6 (55.2)
Operation time, mean (SD), min		266.7 (40.2)	254.4 (46.3)
Intraoperative fentanyl use, mean (SD), μg		611.1 (126.9)	612.5 (155.3)
Time from ICU admission to extubation, mean (SD), min		961.7 (53.8)	962.5 (60.2)
Use of acetaminophen for postoperative analgesia, n (%)		1 (11.1)	1 (12.5)

*SAPB* serratus anterior plane block; *ECOG PS* Eastern Cooperative Oncology Group performance status, *AS* aortic stenosis *ASD* atrial septal defect, *AR* aortic regurgitation, *TR* tricuspid regurgitation, *MR* mitral regurgitation, *TAP* tricuspid annuloplasty, *AVR* aortic valve replacement, *MVP* mitral valve plasty, *SD* standard deviation, *SPPB* Short Physical Performance Battery, *ROM* range of motion, *IQR* interquartile range, *ICU* intensive care unit

**Fig. 2:**
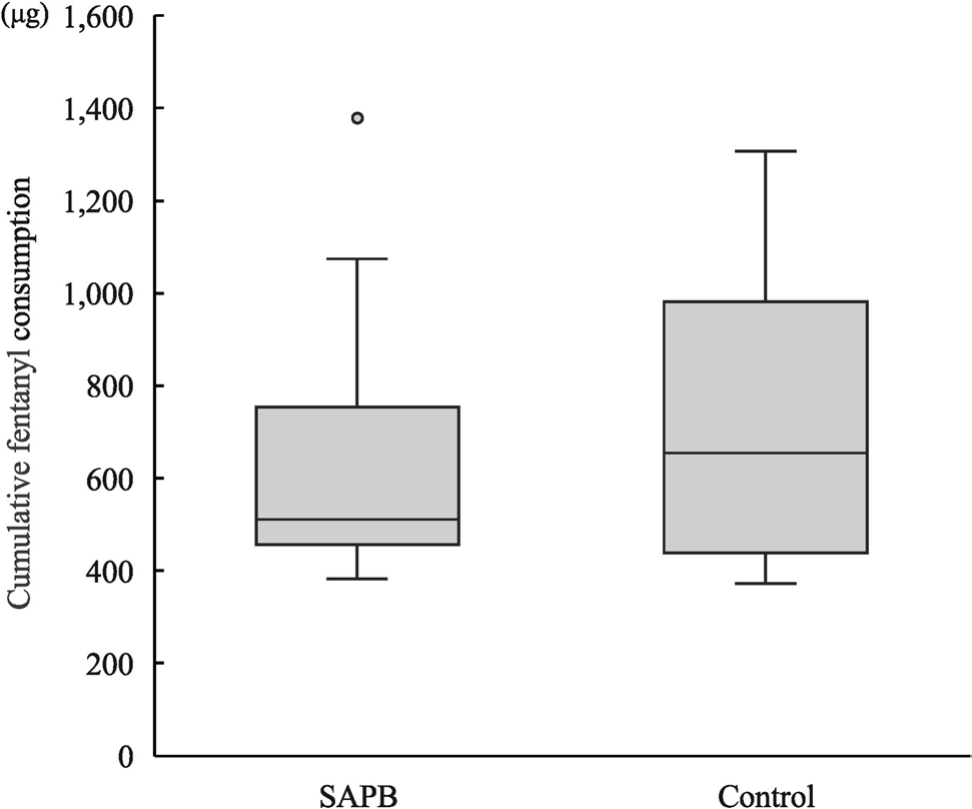
Box plots of cumulative fentanyl consumption up to postoperative day 5. No significant differences were detected between the two groups. *SAPB* serratus anterior plane block

There was no significant difference in cumulative fentanyl consumption up to POD 5 between the two groups (SAPB group, 512 µg [457–753 µg] vs. control group, 654 µg [439–982 µg]; *P* = 0.96) (Fig. 2).

The median NRS scores at rest and on movement showed no significant differences between the two groups. However, the incidence of PONV was significantly lower in the SAPB group compared with the control group (11 vs. 63%; *P* = 0.049) (Table 2). No complications due to PIBI using SAPB were observed in any patient.

**Table 2:** Outcome data

**Outcomes**		**Group**		
		**SAPB (n = 9)**	**Control (n = 8)**	***P* value**
Complications of PIBI with SAPB		0 (0)	0 (0)	
Toxicity of ropivacaine (yes), n (%)		0 (0)	0 (0)	
PONV (yes), n (%)^a^		1 (11)	5 (63)	0.049*
Median NRS score on movement, median (IQR)^b^		3.0 [1.0, 3.0]	3.5 [2.8, 5.0]	0.098
Median NRS score at rest, median (IQR)^b^		1.0 (1.0, 1.0)	1.5 [0.0, 2.3]	0.48
SPPB at the end of study period, n (%)^a^	11	1 (11)	2 (25)	0.58
	12	8 (89)	6 (75)	
6-min walk distance, mean (SD)^c^		411.0 (78.4)	378.3 (103.4)	0.47

*PIBI* programmed intermittent bolus infusion, *SAPB* serratus anterior plane block, *PONV* postoperative nausea and vomiting, *NRS* numerical rating scale, *IQR* interquartile range, *SPPB* Short Physical Performance Battery, *SD* standard deviation

^a^Fisher’s exact test

^b^Mann–Whitney *U* test

^c^Student’s* t*-test

^*^Significant *P* value

**Fig. 3:**
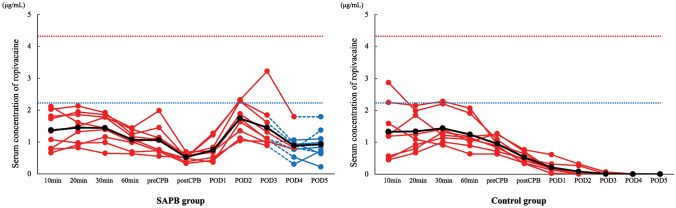
Serum concentration of ropivacaine. Red line charts indicate arterial samples, blue line charts indicate venous samples, and black lines indicate mean data. Red dotted lines indicate the threshold for systemic toxicity of ropivacaine in arterial samples (4.3 µg/mL), and blue dotted lines indicate the threshold in venous samples (2.2 µg/mL). *SAPB* serratus anterior plane block, *CPB* cardiopulmonary bypass, *POD* postoperative day

The serum concentration of ropivacaine in arterial and venous samples did not reach the threshold for systemic toxicity (4.3 µg/mL and 2.2 µg/mL, respectively) in any patient (Fig. 3). The highest individual concentrations were 3.2 µg/mL and 1.8 µg/mL in the arterial and venous samples, respectively, of patients in the SAPB group. No symptoms indicating ropivacaine toxicity were observed.

We observed no significant intergroup differences in the outcomes of rehabilitation, including the SPPB score and 6-min walk distance (Table 2, Supplementary Fig. 1).

## Discussion

PIBI with SAPB did not significantly reduce post-MICS fentanyl consumption, and the PIBI protocol used did not elevate the serum concentration of ropivacaine to the threshold of systemic toxicity.

Ultrasound-guided SAPB was first described by Blanco et al. [9], and the procedure is effective for analgesia in thoracic surgery [13, 14]. SAPB provides analgesia to the anterolateral chest wall by blocking the lateral cutaneous branches of the intercostal nerves. Previous studies have investigated various SAPB methods for improving post-MICS pain management, with varying results. Toscano et al. reported reduced morphine requirements with continuous SAPB during the initial 24 h compared with a control group [5]. Alfirevic et al. showed that the combination of SAPB and pectoralis plane block using a mixture of plain and liposomal bupivacaine did not improve postoperative analgesia after robotically assisted mitral valve repair compared with general anesthesia [6]. Moreover, no prior studies have investigated the efficacy and safety of analgesia using PIBI with SAPB after MICS.

Since we did not observe significant results regarding post-MICS analgesia in this study, we should consider visceral pain, spread of local anesthetics, and surgical sites. First, SAPB is a simple somatic nerve block; visceral analgesia via the spinal sensory afferent neurons may be insufficient. Nociceptive visceral afferents from the visceral pleura and pericardium after thoracotomy are conveyed by the phrenic and vagus nerves [15]. Additionally, chest tube-related pain caused by pleura stimulation, possibly due to the long thoracic, phrenic, thoracodorsal, vagus, and intercostal nerves, might not be sufficiently blocked by SAPB [16]. Thus, SAPB alone may provide insufficient analgesia to treat visceral pain.

Second, a larger volume of local anesthetics may lead to more reliable analgesic coverage. Kunigo et al. found that 40 mL of ropivacaine provided a larger area of sensory loss compared with 20 mL [10]. Although a single 20-mL dose of ropivacaine was administered as postoperative PIBI in this study, it is possible that a larger volume might be needed to obtain an appropriate analgesic area.

Third, as this study involved several MICS surgical procedures, we should consider the various incision sites used in individual procedures. A right anterior thoracotomy was performed for aortic valve surgery, whereas a right mini thoracotomy was performed for mitral valve surgery and atrial septal defect repair. The incision extended to the anteromedial chest wall, which was performed during aortic valve surgery; patients with a small body size might be associated with the anterior cutaneous branches of the intercostal nerves. As SAPB might not adequately cover the anteromedial surgical sites, a combination with other nerve blocks, such as the transversus thoracic muscle plane and parasternal intercostal nerve blocks, should be considered.

As SAPB requires the infusion of a large volume of local anesthetics, toxicity during PIBI must also be acknowledged. To the best of our knowledge, this is the first study to investigate the analgesic safety of PIBI with SAPB. Ropivacaine exhibits a mean terminal half-life of 1.8 ± 0.7 h when administered intravenously and 4.2 ± 1.0 h through epidural administration [17]. Griffiths et al. showed that the mean time of maximum concentration was 35.5 ± 15.7 min after transversus abdominis plane block in patients receiving a cesarean section [18]. Thus, the 6-h interval for PIBI with SAPB in this study could be acceptable. Knudsen et al. showed that the potentially toxic thresholds of ropivacaine were 4.3 µg/mL in arterial samples and 2.2 µg/mL in venous samples [11]. Although the concentration was elevated between PODs 1 and 2 in the SAPB group, it tended to decrease in most cases by POD 3 and did not increase thereafter. The serum concentration of ropivacaine during PIBI remained below the toxic threshold in both arterial and venous samples, and no toxic symptoms were observed in any patient. Our data indicated that serum ropivacaine may not accumulate after repeated PIBI with SAPB; therefore, it may be possible to perform additional nerve blocks or administer a larger volume of local anesthetic in SAPB to obtain adequate analgesic effects.

Although the study was not initially designed to have adequate statistical power to detect differences in the incidence of PONV, it demonstrated a markedly lower incidence of PONV in the SAPB group. PONV is multifactorial, including operative, anesthetic, and patient-specific risk factors [19]; thus, unknown etiologies might contribute to a lower incidence of PONV.

Our study had some limitations. First, we did not evaluate the dermatomal distribution of sensory loss, as we performed the first SAPB followed by catheterization under general anesthesia in all participants. However, SAPB was performed by a single anesthesiologist, and the needle placement was adjusted under ultrasound guidance. Second, one patient in the control group dropped out of the study due to poor pain control and was excluded from the final analysis. As shown in Supplementary Fig. 2, the median NRS score on movement on POD 1, including this patient, was lower in the SAPB group, although the result did not reach statistical significance (SAPB group, 3.0 [2.0–3.5] vs. control group, 4.5 [3.0–5.5]; *P* = 0.051). The withdrawal of this patient from the intervention may have affected the results. Third, the sample size was calculated based on limited retrospective data, and the number of analyzed cases was further reduced to 17 due to dropout. Along with a smaller effect size than originally anticipated and considerable interindividual variability in fentanyl consumption, these factors may have lowered the statistical power and increased the risk of a Type II error, potentially contributing to the absence of statistically significant differences. Fourth, the blood samples for the serum concentration of ropivacaine were collected from the indwelling arterial catheter until its removal, after which samples were taken via venipuncture. We designed this to avoid invasive procedures. As the day of arterial catheter removal differed among individual patients, our data might not accurately reflect the accumulation of ropivacaine. Fifth, the symptoms of ropivacaine toxicity might have been masked under general anesthesia and the use of sedatives until extubation.

In conclusion, PIBI with SAPB showed no significant improvement in cumulative fentanyl consumption or pain scores after MICS. However, the serum concentration of ropivacaine in patients who underwent PIBI with SAPB were clearly below the toxicity threshold, and no complications or adverse events due to this technique were observed. Further investigations should be conducted to compare the multiple approaches of PIBI with SAPB regarding the volume of local anesthetics and their combination with other nerve blocks.

## Supplementary Information

Below is the link to the electronic supplementary material.

Supplementary file 1 (PDF 202 KB)

## Data Availability

The data that support the findings of this study are not openly available due to reasons of sensitivity and are available from the corresponding author upon reasonable request.
