# Supplementary material for: Analgesic efficacy and serum ropivacaine concentration of postoperative programmed intermittent bolus infusion with serratus anterior plane block in patients undergoing minimally invasive cardiac surgery: A randomized, double-blind, controlled trial
Source: J Anesth. 2025 Jul 1;39(6):940–7. doi: 10.1007/s00540-025-03536-4 (PMC12647298; doi:10.1007/s00540-025-03536-4)
Supplement: Supplementary file 1 — (PDF 202 KB) [file 540_2025_3536_MOESM1_ESM.pdf]

Journal of Anesthesia

Analgesic efficacy and serum ropivacaine concentration of postoperative programmed intermittent bolus infusion with serratus anterior plane block in patients undergoing minimally invasive cardiac surgery: A randomized, double-blind, controlled trial

Yuna Sato, Michio Kumagai, Yu Kaiho, Shigekazu Sugino, Tomohiro Sekine, Masataka Taguri, Hiroshi Inoue, Jun Ito, Yu Sato, Toshihiro Sato, Masamitsu Maekawa, Masanori Yamauchi

Corresponding author: Yuna Sato, MD, PhD

E-mail: y0u1n0a4@gmail.com

Supplementary Table 1. MS/MS parameters in selected reaction monitoring analysis for simultaneous quantification

No	Compound	Target	Q1 (*m/z*)	Q3 (*m/z*)	DP (V)	EP (V)	CE (V)	CXP (V)
1	Ropivacaine	Analyte	275	126	76	4	33	24
2	Ropivacaine-d7	IS	282	133	46	4	31	6

MS/MS, tandem mass spectrometry; CE, collision energy; CXP, collision cell exit potential; DP, deculturation potential; EP, entrance potential; IS, internal standard.

Supplementary Figures

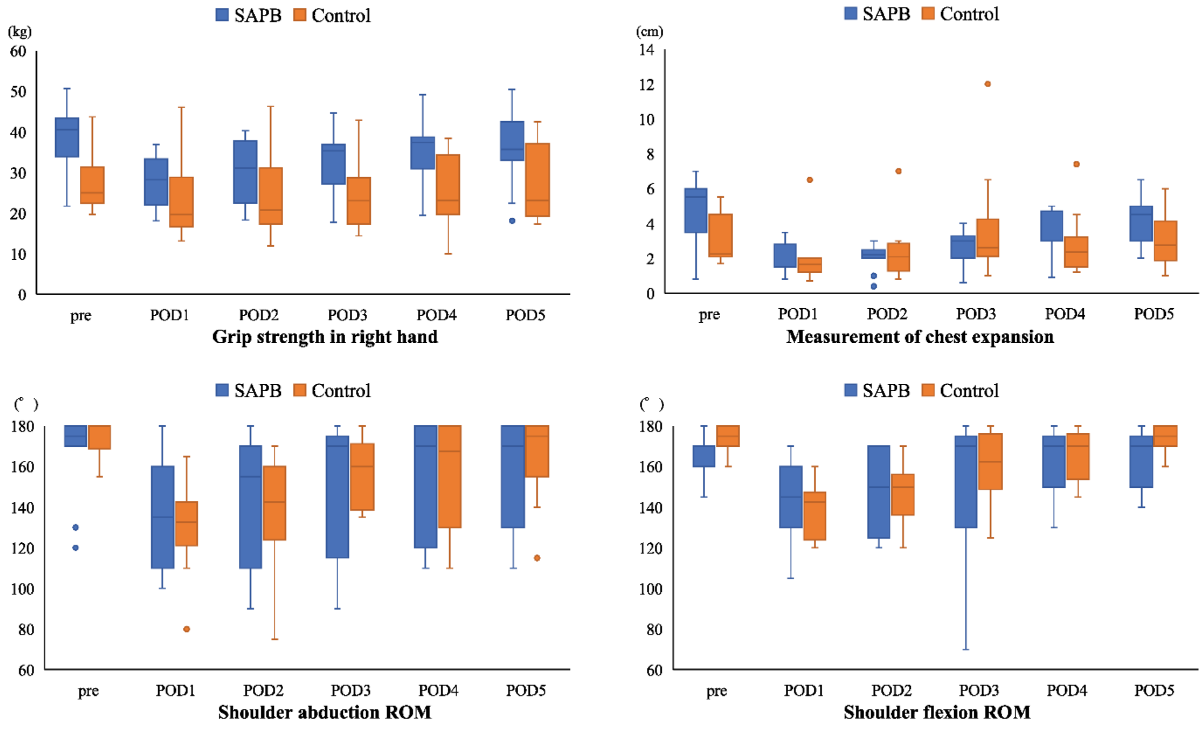

Supplementary Figure 1. Evaluation of Rehabilitation. No significant intergroup differences were observed for any of the outcomes. SAPB, serratus anterior plane lock; POD, postoperative day; ROM, range of motion

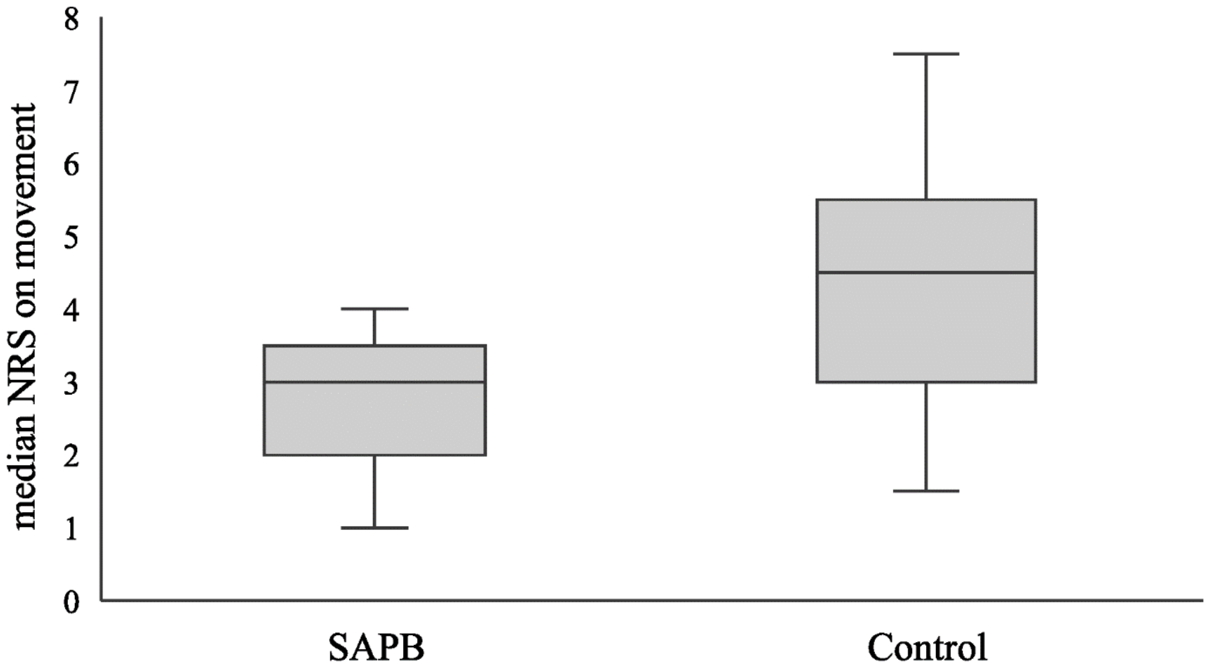

Supplementary Figure 2. Box plots of the median NRS score on movement on POD 1 including the patient excluded from the final analysis due to poor pain control. NRS, numerical rating score; POD, postoperative day
